# Supplementary figures and images for: Macular Choroidal Thickness in Keratoconus: Systematic Review and Meta-Analysis of Current Evidence
Source: Diagnostics (Basel). 2025 Sep 19;15(18):2394. doi: 10.3390/diagnostics15182394 (PMC12469044; doi:10.3390/diagnostics15182394)

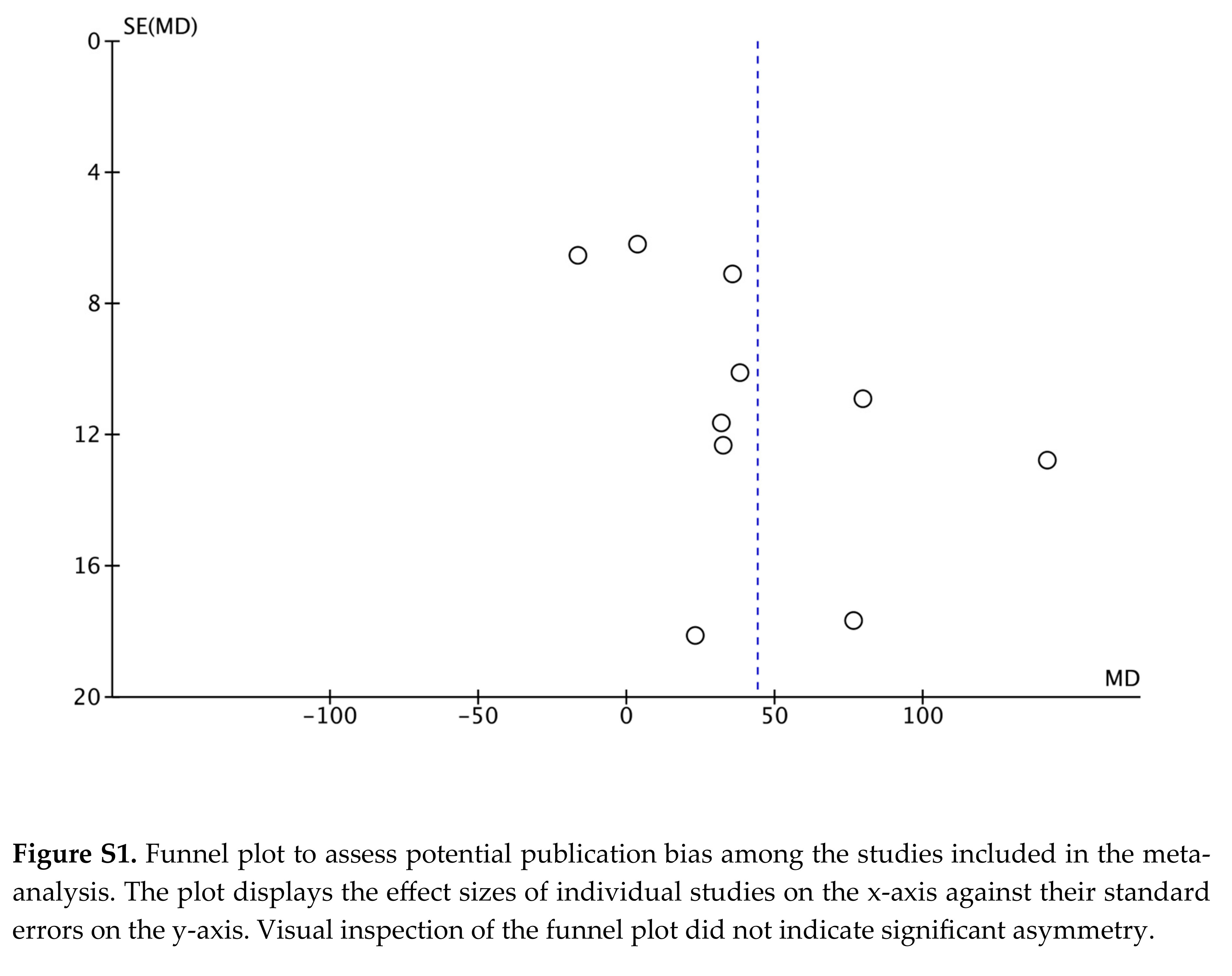

Supplement: Supplementary file 1 [file diagnostics-15-02394-s001.zip › Figure S1.tif]
